# Cardioprotective effects of semaglutide on isolated human ventricular myocardium

**DOI:** 10.1002/ejhf.3644

**Published:** 2025-03-19

**Authors:** Thomas Krammer, Maria J. Baier, Philipp Hegner, Tilman Zschiedrich, David Lukas, Matthias Wolf, Christian Le Phu, Vanessa Lutz, Katja Evert, Kostiantyn Kozakov, Jing Li, Andreas Holzamer, Lars S. Maier, Zdenek Provaznik, Donald M. Bers, Stefan Wagner, Julian Mustroph

**Affiliations:** ^1^ Department of Internal Medicine II University Hospital Regensburg Regensburg Germany; ^2^ Institute for Pathology University of Regensburg Regensburg Germany; ^3^ Cardiothoracic Surgery University Hospital Regensburg Regensburg Germany; ^4^ Department of Pharmacology University of California Davis CA USA

**Keywords:** Calcium, Heart failure, Human cardiomyocytes, Semaglutide

## Abstract

**Aims:**

Semaglutide, a glucagon‐like peptide‐1 (GLP‐1) receptor agonist, has shown promising effects in reducing cardiovascular events in patients with obesity and heart failure (HF) with preserved ejection fraction (HFpEF) irrespective of concomitant diabetes. However, the exact mechanisms underlying its cardioprotective actions remain unclear. Our study investigates the direct effects of semaglutide on human cardiomyocytes, focusing on calcium (Ca) and sodium (Na) handling and its potential to improve myocardial contractility.

**Methods and results:**

Human left ventricular cardiomyocytes were isolated from non‐failing (NF) hearts, patients with aortic stenosis and a HFpEF‐like phenotype (AS), and those with end‐stage HF with reduced ejection fraction (HFrEF). Late Na current (I_Na_), sarcoplasmic reticulum (SR) Ca leak, and contractility were assessed in isolated cardiomyocytes treated with semaglutide. CaMKII inhibitor autocamtide‐2‐related inhibitory peptide and GLP‐1 receptor antagonist exendin 9–39 (Ex‐9‐39) were used to elucidate signalling pathways. Semaglutide reduced late I_Na_ in AS and HFrEF cardiomyocytes to levels comparable to NF. Additionally, semaglutide decreased diastolic SR Ca leak and improved systolic Ca transients and contractility in AS and HFrEF tissue. These effects were mediated through GLP‐1 receptor agonism and were comparable to CaMKII inhibition. In multicellular preparations, semaglutide differentially improved myocardial contractility in AS and HFrEF in a dose‐dependent manner.

**Conclusion:**

Semaglutide directly modulates ion homeostasis in human cardiomyocytes, reducing proarrhythmic diastolic SR Ca leak and enhancing systolic function, which may explain its observed clinical benefits. These findings provide mechanistic insights into the cardioprotective effects of semaglutide and suggest its potential therapeutic use in HF.

## Introduction

The STEP‐HFpEF study investigating the glucagon‐like peptide‐1 (GLP‐1) receptor agonist semaglutide in patients with heart failure (HF) and preserved ejection fraction (HFpEF) and obesity (body mass index [BMI] ≥30 kg/m^2^) showed larger reductions in symptoms and physical limitations, greater improvements in exercise function, and greater weight loss upon treatment with 2.4 mg per week semaglutide than placebo.^1^ Furthermore, in the SELECT study, 2.4 mg per week semaglutide improved a composite of death from cardiovascular causes, non‐fatal myocardial infarction, or non‐fatal stroke in patients with a BMI of ≥27 kg/m^2^, which appeared to be mainly driven by a reduction of a HF composite endpoint.^2^ The exact mode of action of semaglutide is, however, still unclear. As shown in the STEP‐HFpEF and SELECT trials, semaglutide is highly effective even in a non‐diabetic population, underscoring that the glucose‐lowering action of semaglutide may not be the only important effect. Furthermore, semaglutide remains effective across the BMI spectrum of the participants of the trials,^1^, ^2^ hinting at effects beyond weight reduction. A recent post‐hoc analysis of SELECT indicated consistent reductions of major adverse cardiovascular events regardless of the HF subtype of participants (i.e. HFpEF or HF with reduced ejection fraction [HFrEF]),^3^ but no randomized controlled trial for semaglutide in patients with HFrEF exists. A direct effect of semaglutide on cardiomyocyte contractility and sodium (Na) and calcium (Ca) homeostasis could help explain the consistent effects of semaglutide in diabetic and non‐diabetic patients with comorbidities and in patients at risk for or with existing HF.

The effects of GLP‐1 receptor agonists on the cardiovascular system have been investigated in the past with older substances of that class. For example, liraglutide, a second generation GLP‐1 agonist, showed significant cardioprotective effects after left anterior descending coronary artery occlusion in animals.^4^, ^5^ In another pre‐clinical study, the first generation GLP‐1 receptor agonist exendin‐4 prevented post‐myocardial infarction remodelling by impacting extracellular matrix composition.^6^ Importantly, we and others have proposed that direct effects of GLP‐1 receptor agonists on failing human and murine cardiomyocytes may occur via the inhibition of Ca/calmodulin‐dependent kinase II (CaMKII),^7^, ^8^ caused by stimulation of CASK (which can phosphorylate sites on CaMKII that suppress activation).^8^

However, the landmark improvement of clinical cardiovascular endpoints by semaglutide in comparison to the modest effects of the older compounds exenatide (exendin‐4 derivative) or liraglutide indicates differences in either the targeting or the potency of semaglutide. Furthermore, our recent publications in showing clinically relevant direct effects of empagliflozin^9^ or ranolazine^10^ on the cardiac late Na current (I_Na_) in human and murine cardiomyocytes indicate that a clinically important effector downstream of CaMKII may in fact be late I_Na_, whose inhibition could suffice to improve cardiomyocyte Ca and Na handling.^9^, ^10^, ^11^

We thus performed a comprehensive investigation of semaglutide in isolated ventricular cardiomyocytes and multicellular preparations with a focus on human tissue from patients without HF (non‐failing [NF] cardiomyocytes), patients with aortic stenosis (AS) and a HFpEF‐like phenotype, and patients with end‐stage HF undergoing heart transplantation (HFrEF). We propose that the effects of semaglutide on clinical cardiovascular outcomes in the landmark clinical trials may at least in part be explained by a direct improvement in cardiomyocyte Na and Ca homeostasis (*Graphical Abstract*).

## Methods

An expanded methods section can be found in the online *supplementary material*.

### Study approval

All experiments were approved by local committees. The investigation conforms with the principles outlined in the Declaration of Helsinki. Written informed consent by all patients had been given before tissue donation. Animal procedures were performed in accordance with the Guide for the Care and Use of Laboratory Animals, follow the ARRIVE guidelines, and were approved by regulatory authorities.

### Patient data

Patient data were obtained through perioperative assessment. Baseline characteristics are displayed in *Table* *1*.

**Table 1:** Patient data of left ventricular tissue donors

Patient data	Non‐failing^a^ (*n* = 13)	Aortic stenosis (*n* = 23)	HFrEF (*n* = 10)
Age, years, mean ± SEM	54.92 ± 2.29	66.32 ± 1.62	56.6 ± 1.79
Male sex, %	76.92	78.26	70
EF, %, mean ± SEM	58.55 ± 1.3	60.07 ± 2.66	16.75 ± 1.92
E/E', mean ± SEM	8.43 ± 0.8 (*n* = 11)	17.25 **±** 1.97 (*n* = 14)	N/A
Atrial fibrillation, %	0	4.35	0
aHTN, %	46.15	73.91	50
Diabetes, %	53.85	34.78	20
CKD, %	69.23	21.74	40
eGFR, ml/min, mean ± SEM	49.25 ± 4.66	72.04 ± 4.41	60.5 ± 9.41
Creatinine, mg/dl, mean ± SEM	1.65 ± 0.15	1.12 ± 0.1	1.59 ± 0.35
NT‐proBNP, pg/ml, mean ± SEM	N/A	1240.95 ± 263.2	8555.1 ± 3831

Clinical characteristics of patients, from which tissue was obtained. (*n* = XX) denotes number of patients with available values if divergent from total number of patients.

aHTN, arterial hypertension; CKD, chronic kidney disease; E/E′, quotient from pulsed‐wave Doppler E (mitral valve leaflet tips) and tissue Doppler E′ (septal/lateral position adjacent to mitral valve); EF, ejection fraction; eGFR, estimated glomerular filtration rate; HFrEF, heart failure with reduced ejection fraction; N/A, not available; NT‐proBNP, N‐terminal pro‐B‐type natriuretic peptide; SEM, standard error of the mean.

^a^ Non‐failing left ventricular tissue: graft rejection was excluded histologically for all patients.

### Human cardiac tissue

Left ventricular (LV) myocardium was acquired from hearts explanted from patients in end‐stage HF (HFrEF) that were receiving heart transplant. Additionally, LV samples were acquired from patients undergoing aortic valve replacement for AS with a HFpEF‐like phenotype (New York Heart Association class >1, elevated N‐terminal pro‐B‐type natriuretic peptide and diastolic dysfunction) (*Table* *1*). LV tissue from patients without HF (i.e. NF tissue) was obtained from heart transplanted patients undergoing standard‐of‐care post‐transplant LV endomyocardial biopsy (patients consented to extraction and donation of additional scientific biopsies during the clinical procedure). Graft rejection was excluded for all patients in the clinical biopsies via histology by our expert pathologists.

For human cardiomyocytes, cell isolation was performed from vibratome‐cut myocardial slices as described previously.^12^ Further details can be found in the online *supplementary material*.

### Isolation of murine cardiomyocytes

Isolation of cardiomyocytes was performed as previously described.^13^, ^14^ Wild‐type (WT) C57BL6/J mice (age: 12.63 ± 0.2 weeks; body weight: 26.41 ± 0.38 g; heart weight: 182.3 ± 3.81 mg, all values mean ± standard error of the mean [SEM]) were obtained commercially from Janvier laboratory (Le Genest‐Saint‐Isle, France). Further details on the isolation can be found in the online *supplementary material*.

### Drugs/substances

Semaglutide (Sema, AdipoGen, Liestal, Switzerland) was investigated at concentrations of 50, 100, and 300 nmol/L. Stock solutions of semaglutide were dissolved in DMSO and kept at −20°C. On the day of the experiment, semaglutide was diluted to the final concentrations in Krebs–Henseleit, Tyrode buffer or patch clamp bath solution (see below). For control (vehicle) experiments, equal amounts of DMSO were used. For some experiments, the CaMKII inhibitory peptide autocamtide‐2 related inhibitory peptide (AIP, Enzo, USA, 2 μmol/L) was used in its myristoylated form for better membrane permeability. Similarly, the GLP‐1 receptor antagonist exendin 9–39 (Ex‐9‐39, Hycultec, Germany, 100 nmol/L for cellular experiments, 600 nmol/L for trabeculae) was used for some experiments. For some experiments, H_2_O_2_ was diluted from a 30% H_2_O_2_ solution (Roth, Germany) to a final concentration of 50 μmol/L for cellular experiments, 100 μmol/L for trabeculae. The exposure to substances and the selection of mice for cellular isolation were randomized. Investigators were blinded regarding the investigational substances (e.g. semaglutide). The human tissue experiments were unblinded regarding the aetiology (NF, AS, HFrEF) for legal reasons and due to the clear identifiability of the tissue (e.g. NF biopsies and whole hearts for HFrEF).

### Epifluorescence measurements and calcium spark measurements

Calcium epifluorescence measurements were performed using an epifluorescence setup (IonOptix Corp, Milton, MA, USA) mounted to a Motic AE31 microscope.^15^ Ca sparks were assessed using an LSM 700 confocal microscope (Zeiss, Germany). Details can be found in the online *supplementary material*.

### Muscle strip experiments

Muscle strip experiments were performed as previously reported.^16^ Arrhythmias were assessed using the arrhythmia severity score as previously described.^17^ For further details, see online *supplementary material*.

### Slice culture and force measurements

Human LV myocardial tissue of patients with AS and a HFpEF‐like phenotype or of patients with end‐stage HFrEF undergoing heart transplantation was sliced using a vibratome and then placed in a culturing chamber system for measurement of contraction force as described by Hamers *et al*.^18^ Further details can be found in the online *supplementary material*.

### Patch‐clamp experiments

Late I_Na_ ^19^ and action potentials (APs) and early/delayed afterdepolarizations were recorded as previously described.^20^ Further details can be found in the online *supplementary material*.

### Statistical tests

Data are expressed as mean ± SEM per patient or mouse unless noted otherwise. Normality of data was tested by hierarchical application of D'Agostino–Pearson, Anderson–Darling, or Shapiro–Wilk tests. When suitable mixed‐effects models were used with Holm–Šídák multiple comparisons post‐tests; otherwise, a Kruskal–Wallis test was performed with Dunn's post‐test. *P*‐values of the overall test are referenced in the figure legends, *p*‐values in the figure represent the post‐hoc individual comparisons, unless noted. For two groups without pairing, a Mann–Whitney test was used. For contingency data, the Fisher's exact test was used. Two‐sided *p* < 0.05 was considered significant. Statistics were performed using Graph Pad Prism 10.

## Results

### Semaglutide inhibits late sodium current in human ventricular myocardium

We previously reported that cardiac late I_Na_ is increased in patients with AS with a HFpEF‐like phenotype and that late I_Na_ can be inhibited in human cardiomyocytes by limiting CaMKII activity with empagliflozin^9^ or by direct inhibition of late I_Na_ with ranolazine.^10^ Earlier, we had shown that exendin‐4, a first generation GLP‐1 receptor agonist, can limit stimulatory CaMKII activity that promotes pathological late I_Na_ induction.^8^

**Figure 1:**
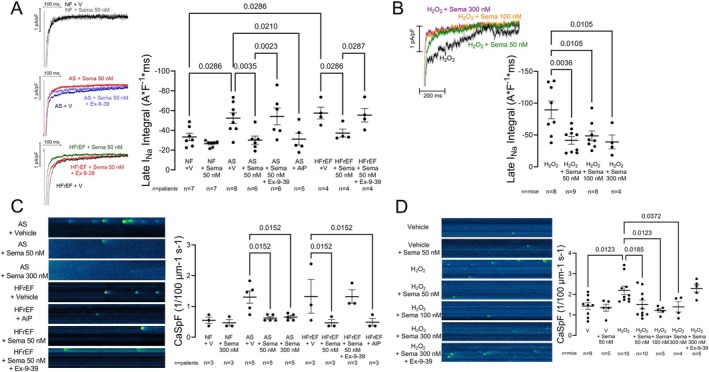
Semaglutide (Sema) reduces late sodium current (I_Na_) and diastolic calcium (Ca) leak in human ventricular cardiomyocytes. (*A*) Original recordings (left panel) and mean data (right panel) of late I_Na_ in human ventricular cardiomyocytes from non‐failing (NF), aortic stenosis (AS), and heart failure with reduced ejection fraction (HFrEF) patients (*n* = patients). In cardiomyocytes from NF patients, no relevant late I_Na_ could be observed upon vehicle control (V), which was consequently unaltered by Sema. In contrast, significant late I_Na_ was observed in cardiomyocytes from patients with AS and a HFpEF‐like phenotype, which was reduced to NF levels already at 50 nmol/L Sema. This effect was comparable to CaMKII inhibition with 2 μmol/L autocamtide‐2 related inhibitory peptide (AIP). The inhibitory effect of Sema was abrogated by glucagon‐like peptide‐1 (GLP‐1) receptor antagonism with exendin‐9–39 (Ex‐9–39). Similarly, significant late I_Na_ was observed in cardiomyocytes from patients with end‐stage heart failure, which was inhibited by 50 nmol/L Sema. Ex‐9–39 also prevented the inhibitory effect of Sema on late I_Na_ in HFrEF cardiomyocytes. Data tested with a mixed‐effects model (overall *p = 0.0002). *P*‐values in figure represent Holm–Sidak post‐hoc *p*‐values. (*B*) Original recordings (left panel) and mean data (right panel) of late I_Na_ in murine cardiomyocytes from wild‐type C57BL6 mice upon H_2_O_2_ stress (*n* = mice). The H_2_O_2_‐induced enhancement of late I_Na_ was inhibited by 50, 100 and 300 nM Sema independent of the concentration. Data tested with a mixed‐effects model (overall *p = 0.0040). (*C*) Original confocal line scan recordings of Ca sparks (left panel) and mean data of Ca spark frequency (CaSpF, right panel) in human ventricular cardiomyocytes from NF, AS and HFrEF patients (*n* = patients). In NF cardiomyocytes, expectedly, no significant occurrence of Ca sparks was observed, and Sema did not affect Ca spark frequency. In contrast, significant occurrence of Ca sparks was observed in human AS and HFrEF cardiomyocytes, which were reduced to NF levels already by 50 nmol/L Sema, which was similar to the effect of CaMKII inhibition in HFrEF with 2 μmol/L AIP. In AS cardiomyocytes, no additional (or inverse) effect was observed upon 300 nmol/L Sema compared to 50 nmol/L. In contrast, GLP‐1 receptor antagonism with Ex‐9–39 abrogated the effect of simultaneous Sema exposure. Data tested with a mixed‐effects model (overall *p = 0.0039). (*D*) Original confocal line scan recordings of Ca sparks (left panel) and mean data of CaSpF (right panel). H_2_O_2_ stimulation was required to induce relevant sarcoplasmic reticulum Ca sparks in murine wild‐type cardiomyocytes and for a notable suppression of sarcoplasmic reticulum Ca sparks by Sema. Sema showed efficacy at 50, 100, and 300 nmol/L, but even at 300 nmol/L could be suppressed by GLP‐1 receptor antagonism with Ex‐9–39. Data tested using a mixed‐effects model (overall *p = 0.0027). (*A–D*) *P*‐values represent Holm–Sidak post‐hoc *p*‐values. Data shown as mean ± standard error of the mean per patient (*A* + *C*) or mouse (*B* + *D*).

Given the central role of late I_Na_ for contractile dysfunction and arrhythmias in human HF,^15^, ^21^, ^22^, ^23^, ^24^ we hypothesized that semaglutide could limit late I_Na_ by a direct cardiomyocyte effect. As expected, in human NF ventricular cardiomyocytes, late I_Na_ was low, and was not decreased further by semaglutide (*Figure* *1A*). Cardiomyocytes from patients with AS and a HFpEF phenotype or patients with end‐stage HF (HFrEF) both exhibited elevated late I_Na_ compared to NF patients (*Figure* *1A*). Semaglutide, even at 50 nmol/L, reduced late I_Na_ in both AS and HFrEF to values comparable to NF cardiomyocytes (*Figure* *1A*). For comparison, the CaMKII inhibitor AIP reduced late I_Na_ to a similar extent in AS (*Figure* *1A*). Moreover, simultaneous exposure of either AS or HFrEF cardiomyocytes to semaglutide plus the GLP‐1 receptor antagonist Ex‐9–39 prevented the inhibitory effect of semaglutide on late I_Na_.

The clinically observed plasma levels of semaglutide may vary depending on ethnicity, gender, duration of exposure and other factors, but values of ~50 to ~96 nmol/L are typically reported for the approved 1.0–2.4 mg doses of semaglutide.^25^, ^26^, ^27^ The efficacy of clinically relevant concentrations of semaglutide on late I_Na_ was assessed using WT murine cardiomyocytes stimulated with H_2_O_2_ to induce late I_Na_ ^9^ and incubated with 5, 10, 50, 100, or 300 nM semaglutide for 30 min. While 5 and 10 nM semaglutide had no effect on late I_Na_ (data not shown), the clinically relevant concentrations of 50, 100, and 300 nM semaglutide inhibited late I_Na_ to similar extents (*Figure* *1B*). We thus decided to use 50–300 nM semaglutide for further experiments.

### Semaglutide attenuates diastolic calcium leak in human cardiomyocytes

Diastolic sarcoplasmic reticulum (SR) Ca leak can reduce contractile function due to depletion of SR Ca content and cause diastolic dysfunction (by elevating diastolic [Ca]_i_).^28^, ^29^, ^30^ We assessed SR Ca leak in human myocardium by measuring SR Ca sparks. Basal SR Ca spark frequency (CaSpF; a robust parameter for assessment of Ca leak^20^) was low in human NF ventricular cardiomyocytes, and was not lowered further by even 300 nmol/L semaglutide (*Figure* *1C*; a certain low CaSpF is normal due to the spontaneous diastolic opening probability of cardiac ryanodine receptors^28^, ^31^). SR CaSpF was elevated in ventricular cardiomyocytes from patients with AS or HFrEF (*Figure* *1C*). Semaglutide, even at 50 nmol/L, fully returned SR Ca spark frequency to the NF myocyte level. Again, we saw no further benefit when 300 nmol/L semaglutide was used in AS myocytes (*Figure* *1C*). In human HFrEF, 50 nmol/L semaglutide was comparable to inhibition of CaMKII with AIP 2 μmol/L (*Figure* *1C*). Moreover, the GLP‐1 receptor antagonist Ex‐9–39 was able to abrogate the semaglutide‐induced suppression of CaSpF (*Figure* *1C*).

To evaluate the hypothesis that a pathological stimulus (in human remodelling due to AS or HFrEF) is required for the agency of semaglutide, we investigated WT murine cardiomyocytes exposed to H_2_O_2_.^9^ Basal Ca spark frequency in mouse is higher than in human, but at baseline 50 nM semaglutide had (expectedly) no effect. However, when mouse CaSpF was promoted by H_2_O_2_ exposure, 50–300 nM semaglutide reduced CaSpF to the basal level (*Figure* *1D*). Furthermore, Ex‐9–39 abrogated the semaglutide‐induced suppression of SR Ca spark frequency, even at 300 nmol/L semaglutide (*Figure* *1D*).

Additionally, we wanted to investigate if the suppression of late I_Na_ and SR Ca sparks by semaglutide can suppress cellular arrhythmogenesis.^10^, ^32^, ^33^, ^34^, ^35^, ^36^ In murine ventricular cardiomyocytes, exposure to H_2_O_2_ increased the percentage of myocytes showing early and delayed afterdepolarizations, which are typically mediated by late I_Na_ and SR Ca leak, respectively (online supplementary *Figure* *S1*). Semaglutide at 50 and 300 nmol/L effectively suppressed both drivers of cellular arrhythmogenesis (online supplementary *Figure* *S1A*). Moreover, in murine ventricular trabeculae, H_2_O_2_ also promoted arrhythmias, as assessed by an established arrhythmia score (online supplementary *Figure* *S1B*).^17^ Semaglutide (at 50 and 300 nmol/L) effectively suppressed arrhythmias in the murine trabeculae upon H_2_O_2_.

### Semaglutide improves systolic calcium transients dependent on glucagon‐like peptide‐1 receptor expression levels

**Figure 2:**
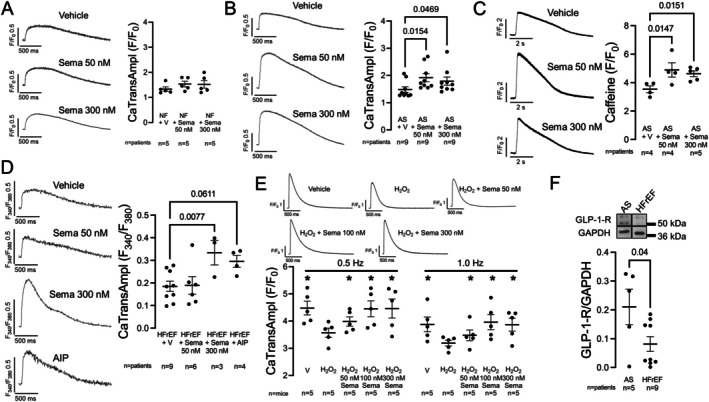
Semaglutide (Sema) improves systolic calcium (Ca) transients dependent on glucagon‐like peptide‐1 receptor (GLP‐1‐R) expression levels. (*A*) Original recordings (left) and mean Ca transient amplitudes (CaTransAmpl) (right) of human ventricular cardiomyocytes from non‐failing (NF) patients. Expectedly, Sema did not show a significant positive inotropic effect. Data tested with a mixed‐effects model (overall *p* = NS). (*B*) Original recordings (left) and mean CaTransAmpl (right) of human ventricular cardiomyocytes from patients undergoing aortic valve replacement due to aortic stenosis (AS) and a heart failure with preserved ejection fraction‐like phenotype. In cardiomyocytes from AS, Sema improved CaTransAmpl. Data tested with a mixed‐effects model (overall *p = 0.0219). (*C*) Original recordings (left) and mean caffeine‐induced Ca amplitudes (right) as a marker of sarcoplasmic reticulum Ca content of human ventricular cardiomyocytes from AS. Sema improved sarcoplasmic reticulum Ca content comparably at 50 and 300 nmol/L. Data tested with a mixed‐effects model (overall *p = 0.0160). (*D*) Original recordings (left) and mean CaTransAmpl (right) of human ventricular cardiomyocytes from patients with heart failure with reduced ejection fraction (HFrEF). Sema did not improve CaTransAmpl at 50 nmol/L but was significant and effective at 300 nmol/L. Sema at 300 nmol/L showed comparable efficacy to CaMKII inhibition with 2 μmol/L autocamtide‐2‐related inhibitory peptide (AIP) (AIP showed borderline non‐significance in the post‐test, but a clear numerical effect). Data tested with a mixed‐effects model (overall *p = 0.0204). (*E*) Original recordings (upper panel) and mean CaTransAmpl (lower panel) of murine ventricular cardiomyocytes exposed H_2_O_2_‐stress ± Sema. Expectedly, H_2_O_2_ depressed CaTransAmpl. While numerical values differed between 50 and 300 nmol/L, all concentrations were effective and significant in ameliorating the pathological H_2_O_2_ stimulus. Data tested with a multifactor mixed‐effects model (overall *p for factor drug/intervention = 0.0152, *p for factor frequency = 0.001). For better readability, the full test results and post‐test values can be found in online supplementary *Figure* *S2A*. (*F*) Western blot showing reduced expression of the GLP‐1‐R in ventricular myocardium from HFrEF compared to AS (tested with Student's *t*‐test). (*A–F*) Data shown as mean ± standard error of the mean per patient (*A–D*, *F*) or mouse (*E*). *P*‐values in figures (*A–E*) represent Holm–Sidak post‐hoc *p*‐values (shown if main test indicated significance). GAPDH, glyceraldehyde‐3‐phosphate‐dehydrogenase (housekeeping protein); V, vehicle control.

As the suppression of SR Ca leak and late I_Na_ upon semaglutide could translate into improvement of systolic Ca release, we assessed electrically stimulated Ca transients in human cardiomyocytes. In human NF cardiomyocytes, semaglutide at 50 or 300 nmol/L did not significantly alter Ca transient amplitudes (*Figure* *2A*), consistent with the low SR Ca leak (*Figure* *1C*). However, in remodelled cardiomyocytes from patients with AS (and a HFpEF‐like phenotype), 50–300 nmol/L semaglutide increased Ca transient amplitudes (*Figure* *2B*). To investigate if improvement of Ca transients by semaglutide involved an increase in SR Ca content (which was reduced by elevated Ca sparks), we next assessed caffeine‐induced Ca transients to estimate SR Ca content in human AS cardiomyocytes. Indeed, both 50 and 300 nmol/L semaglutide increased SR Ca content (*Figure* *2C*).

In human HFrEF cardiomyocytes, 50 nmol/L semaglutide did not increase systolic Ca transients significantly (*Figure* *2D*). However, both 300 nmol/L semaglutide or 2 μmol/L of the CaMKII inhibitor AIP were able to improve human HFrEF Ca transients (*Figure* *2D*).

We therefore exposed murine cardiomyocytes to 50 μM H_2_O_2_ as a model of pathological stress induction. As expected, H_2_O_2_ decreased systolic Ca transient amplitudes compared to vehicle control (*Figure* *2E*). While 50 nmol/L semaglutide did not improve Ca transients as much as 100 and 300 nmol/L semaglutide, all concentrations improved Ca transients (*Figure* *2E*). We thus hypothesized that pathological remodelling in human HFrEF might alter GLP‐1 receptor expression levels, explaining the different dose–response observed in human AS versus HFrEF. Correspondingly, in Western blots of human LV myocardium from AS and HFrEF, we observed lower GLP‐1 receptor expression levels in HFrEF versus AS (*Figure* *2F*).

### Semaglutide improves contractility in human ventricular myocardium

**Figure 3:**
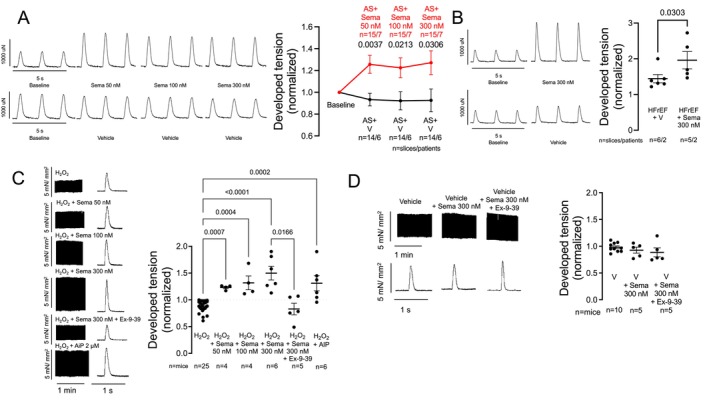
Semaglutide (Sema) improves contractility in human ventricular multicellular preparations. (*A*) Original recordings of contractions (force development, left) and normalized developed tension (right) of human left ventricular muscle slices from patients undergoing aortic valve replacement due to aortic stenosis (AS) and a heart failure with preserved ejection fraction‐like phenotype. Sema improved developed tension at 50, 100, and 300 nmol/L compared to vehicle control (V) (in separate slices from the same patients to exclude confounding rundown effects). Data tested using a multifactor mixed‐effects model (overall *p for factor drug/intervention = 0.0145, *p* for factor time = NS). Data shown as mean ± standard error of the mean (SEM) of individual slices (due to the normalization to baseline and comparison to corresponding vehicle slices from the same patients). (*B*) Original recordings of contractions (force development, left) and normalized developed tension (right) of human left ventricular muscle slices from patients with heart failure with reduced ejection fraction (HFrEF). Sema improved developed tension at 300 nmol/L. Data tested using a Mann–Whitney test. Data shown as mean ± SEM of individual slices (due to the normalization to baseline and comparison to corresponding vehicle slices from the same patients). (*C*) Original recordings of contractions (force development, left) and normalized developed tension (right) of isolated murine ventricular trabeculae exposed to H_2_O_2_ stress. H_2_O_2_ was expectedly negatively inotropic compared to the baseline measurement of the same trabeculae. Sema exerted a positive inotropic effect at 50, 100, and 300 nmol/L that was similar to CaMKII inhibition with 2 μmol/L autocamtide‐2‐related inhibitory peptide (AIP). Glucagon‐like peptide‐1 receptor antagonism with exendin‐9–39 (Ex‐9–39) abrogated the inotropic effect of Sema even at 300 nmol/L. Data shown as mean ± SEM per mouse. Data tested using a Kruskal–Wallis test (overall *p < 0.0001). (*D*) Original recordings of contractions (force development, left) and normalized developed tension (right) of isolated murine ventricular trabeculae exposed to vehicle control, Sema, or Sema + Ex‐9–39. Expectedly, without a pathological stimulus such as H_2_O_2_, no significant effect of Sema on developed tension was observed (and consequently no antagonization of such an effect by Ex‐9–39 could be detected). Data shown as mean ± SEM per mouse. Data tested using a Kruskal–Wallis test (overall *p* = NS). (*A–D*) *P*‐values represent Holm–Sidak (*A* + *B*) or Dunn (*C* + *D*) post‐hoc *p*‐values (shown if main test indicated significance).

To analyse whether the improved Ca transient amplitudes upon semaglutide translate into improved contractility in human ventricular myocardium, we used isolated human ventricular slices using a Myo dish system to record force. In these multicellular preparations of human LV AS samples, semaglutide improved developed tension at 50, 100, and 300 nmol/L compared to vehicle control (exposed slices from the same patients) (*Figure* *3A*). Furthermore, in limited experiments from human LV slices from HFrEF, 300 nmol/L semaglutide also improved developed tension (*Figure* *3B*).

We also evaluated semaglutide in murine ventricular trabeculae exposed to H_2_O_2_. The H_2_O_2_ exposure was negatively inotropic compared to baseline of the same trabeculae (not shown). Semaglutide improved developed tension at all concentrations (50, 100, 300 nmol/L) and AIP produced similar effects (*Figure* *3C*). The inotropic effect of semaglutide was also abrogated by simultaneous GLP‐1 receptor antagonism with Ex‐9–39 (*Figure* *3C*). Moreover, the inotropic effect of semaglutide was not observed in vehicle‐treated mouse trabeculae, with or without Ex‐9–39 (*Figure* *3D*).

## Discussion

In our investigation of semaglutide in human and murine ventricular myocardium, we show improved contractility in isolated cardiomyocytes and multicellular preparations upon clinically observed concentrations of semaglutide. The improved contraction was reflected in increased Ca transient amplitudes upon acute treatment with semaglutide. Exposure of cardiomyocytes and trabeculae to Ex‐9–39 abrogated the effects of semaglutide, therefore indicating a direct agonism of semaglutide on the GLP‐1 receptor. In our data set, semaglutide was able to suppress late I_Na_ and SR Ca sparks to a similar extent as the direct CaMKII inhibitor AIP, which raises the question of the potential signalling pathway. In pancreatic islet cells, the CaMKII regulatory protein CASK can be stimulated by protein kinase A (PKA) downstream of the GLP‐1 receptor.^37^ We have previously shown that the GLP‐1 receptor agonist exendin‐4 can promote inhibitory CaMKII autophosphorylation in cardiomyocytes via CASK.^8^ However, CASK is also an important scaffolding protein^8^, ^38^, ^39^, ^40^, ^41^, ^42^ and its localization likely affected by PKA phosphorylation,^38^, ^39^, ^41^ therefore alternative explanations to a direct modulation of CaMKII activity by CASK exist. Our data on the efficacy of semaglutide in H_2_O_2_‐exposed murine cardiomyocytes could also implicate reactive oxygen species, which can activate CaMKII by oxidation.^43^ Semaglutide has previously been shown to ameliorate reactive oxygen species levels in the serum and cardiac tissue of mice with obesity, possibly related to anti‐inflammatory effects on neutrophils.^44^ Semaglutide could also directly affect cardiomyocyte energy homeostasis, for example, by protecting the structural integrity and function of mitochondria.^45^ Notably, the observed reduction in SR Ca leak might also attenuate mitochondrial dysfunction in HF.^46^, ^47^ Furthermore, semaglutide has been reported to improve glycaemic control in a unique mouse model of HFpEF in comparison with a pair‐fed control group.^48^ In this HFpEF mouse model, semaglutide, in proteome analyses and single cell sequencing, led to upregulation of signalling pathways related to improved mitochondrial function and contraction,^48^ whereas antiapoptotic signalling and an influence on T‐cell regulation and endothelial function was also observed, which, at least in multicellular preparations, could affect cardiomyocyte function.

Differences in myocardial GLP‐1 receptor expression may explain the varying dose–response of Ca transients to semaglutide between the human AS patients with a HFpEF‐like phenotype, which exhibited higher GLP‐1 receptor expression, and the end‐stage HFrEF patients. This could point towards an importance of the degree of remodelling for the efficacy of semaglutide. While our data do not delineate the exact mechanism of GLP‐1 receptor downregulation in HFrEF, GLP‐1 receptor downregulation has been reported in other tissues chronically exposed to GLP‐1, high glucose levels, or ischaemia.^49^, ^50^, ^51^, ^52^ Interestingly, as activation of the renin–angiotensin–aldosterone system is highly prevalent in HF, data from other organs indicating a stimulation of GLP‐1 release upon angiotensin II^53^ could indicate a novel feedback loop worth investigating in the future.

Our data may provide a rationale for the clinical investigation of high‐dose semaglutide in subsets of HF patients and could potentially serve to reconsider the lower doses (i.e. 1 mg/s.c. per week) approved for the management of diabetic patients if they simultaneously suffer from HF, especially HFrEF. However, while the effects of semaglutide on clinical outcomes were consistent across the spectrum of ejection fractions in the SELECT and STEP‐HFpEF trials,^1^, ^2^ these trials were not specifically designed to investigate the effects of semaglutide in HF patients across a broad range of LV ejection fractions and included only a low number of HFrEF patients. No results from dedicated randomized controlled trials for semaglutide in HFrEF exist. As reviewed by Ferreira *et al*.,^54^ at least the ‘older’ GLP‐1 receptor agonists liraglutide and exenatide showed numerical trends in HFrEF patients towards higher hospitalization rates in the FIGHT (liraglutide) and EXSCEL (exenatide) trials, more arrhythmias (FIGHT and LIVE trials for liraglutide), or even all‐cause death (FIGHT trial). The positive inotropic effects observed by us upon semaglutide may thus raise caution in HFrEF, where inotropic therapy in acute decompensated HF is typically associated with adverse outcome. However, those consequences are usually associated with higher energy expenditure for a given inotropic effect. While such energetic balance is not the topic of our current investigations, if the inotropic response is due largely to a decreased SR Ca leak and higher SR Ca content, that would be energy conserving (i.e. not wasting ATP in the futile SR Ca‐ATPase pumping against elevated leak), where SERCA2a activity is a major determinant of energy consumption in the failing heart.^55^ This is also supported by our human NF data, where no pathological SR Ca leak or late I_Na_ induction are observed, and consequently no relevant inotropic response can be measured. Additionally, our murine data with H_2_O_2_‐induced stress support the notion that the efficacy of semaglutide depends on pathological stimuli and/or cardiac remodelling. Interestingly, the strong reduction in late I_Na_ and SR Ca sparks, both known inductors of triggered activity in the form of early and delayed afterdepolarizations,^30^, ^36^, ^56^, ^57^ may also translate into reduced cellular arrhythmogenesis in our data set.

One limitation of our investigation may be the relatively short‐term exposure of the human myocardium to semaglutide. However, the focus of this study was to investigate acute/short‐term semaglutide effects in human ventricular myocardium. Moreover, the extensive human data set is also a strong point of our study in allowing us to assess direct cardiac myocyte consequences, without the longer‐term exposure that complicates interpretations by significant reverse remodelling (both non‐cardiac and cardiac) upon semaglutide therapy. The patient collective was predominantly male (>70% in NF, AS, and HFrEF), but this represents the prevalence of the underlying diseases leading to tissue donations (and we were able to include ~20% female patients in all groups). Similar to the sodium–glucose cotransporter 2 inhibitors, the ‘definite’ mode of action of semaglutide in obesity, HF, and diabetes will likely remain an open question in the near future. It may very well be the case that the astounding clinical effects in a variety of patient populations with and without diabetes, with and without HF are the result of pleiotropic action on multiple organs and also depend on remodelling and the presence of clinical risk factors. However, our extensive human data set supports the notion of a strong direct effect on the myocardium via the GLP‐1 receptor. Furthermore, our data may support high‐dose investigation of semaglutide in diabetic patients with HF and may provide a rationale for the investigation of semaglutide in HFrEF.

## Supporting information


**Figure S1.** Semaglutide effectively suppresses arrhythmias in murine ventricular cardiomyocytes and multicellular trabeculae. (A) Original registrations of action potential measurements in wildtype murine ventricular cardiomyocytes with an arrhythmia protocol upon vehicle control (V), H_2_O_2_, H_2_O_2_ + 50 nmol/L semaglutide, and H_2_O_2_ + 300 nmol/L semaglutide (top). Percentage of cells showing early afterdepolarizations (EADs, lower left) or delayed afterdepolarizations (DADs, lower right). Expectedly, incidence of EADs and DADs was low in healthy WT cardiomyocytes and required a pathological stimulus (H_2_O_2_). Semaglutide effectively lowered the percentage of cells displaying EADs or DADs upon H_2_O_2_ and there was no clear difference between 50 or 300 nmol/L semaglutide. Data shown as percentage of cells displaying EADs or DADs. Data tested using Fisher's exact test. (B) Original murine ventricular trabeculae registrations upon baseline (vehicle control), H_2_O_2_ + Ca stimulus (to provoke arrhythmias), H_2_O_2_ + Ca + 50 nmol/L semaglutide, and H_2_O_2_ + Ca + 300 nmol/L semaglutide (left). Mean ± SEM of the arrhythmia score per mouse (right). Expectedly, the arrhythmia score was low at baseline in multicellular murine ventricular preparations and a pathological stimulus (H_2_O_2_ + Ca) was required to induce arrhythmogenesis. Semaglutide at 50 or 300 nmol/L effectively reduced the arrhythmia score upon simultaneous H_2_O_2_ + Ca exposure. Data shown as mean ± SEM per mouse. Data tested using a mixed‐effects model (overall *p < 0.0001). AP, action potential; DAD(s), delayed afterdepolarizations; EAD(s), early afterdepolarization(s); Sema, semaglutide; V, vehicle control.

**Figure S2.** Full statistical reporting for *Figure* *2E*. (A) For better readability of *Figure* *2E*, the full statistical reporting of the multiparameter mixed‐effects model including the Holm‐Sidak post‐test can be found in (B).

Supplemental Methods
