# Supplementary figures and images for: Cardioprotective effects of semaglutide on isolated human ventricular myocardium
Source: Eur J Heart Fail. 2025 Mar 19;27(7):1315–25. doi: 10.1002/ejhf.3644 (PMC12370581; doi:10.1002/ejhf.3644)

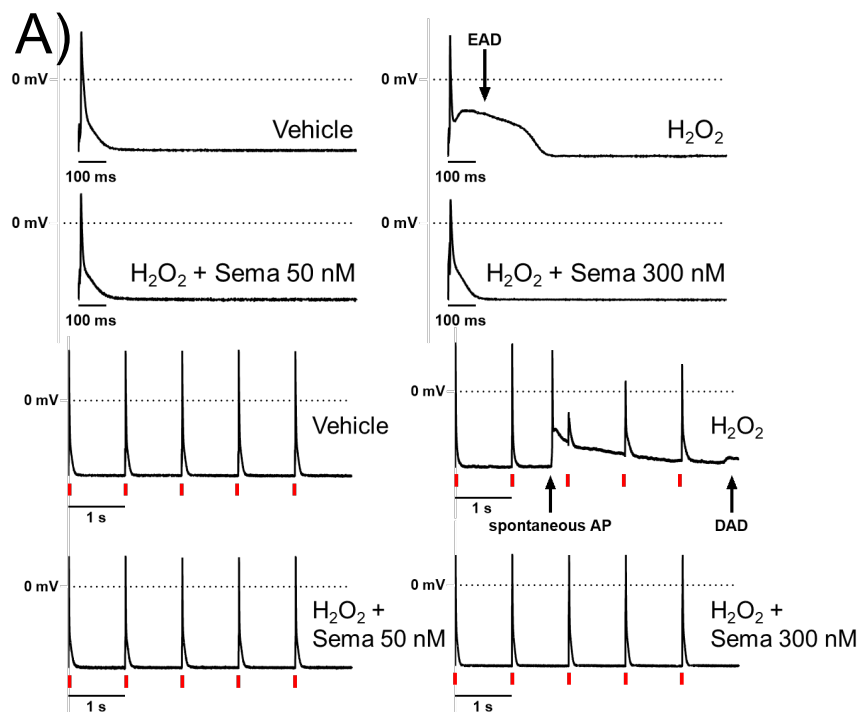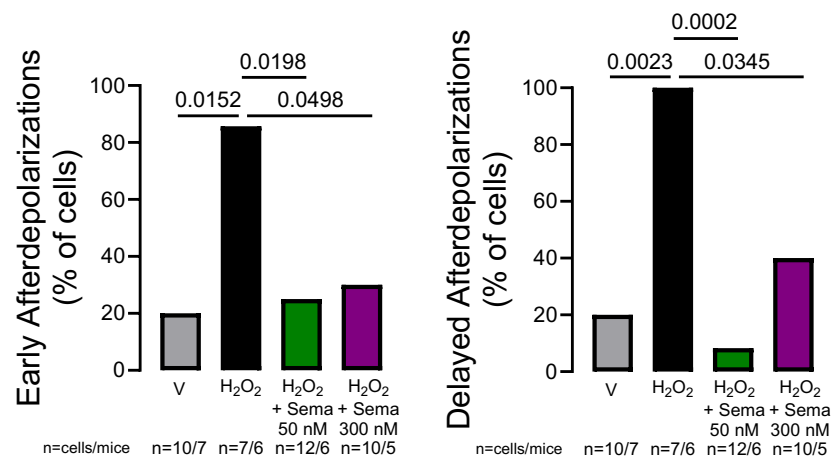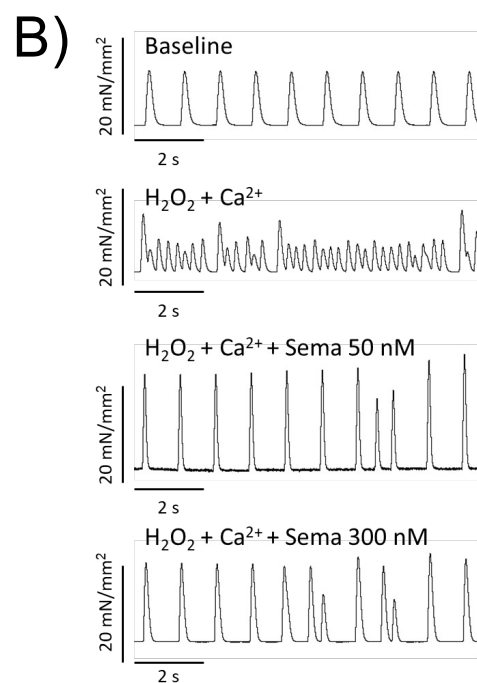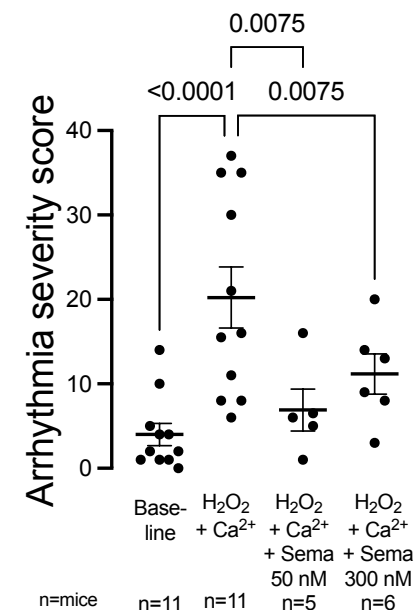

**Supplementary figure S1**

Supplement: Supplementary file 1 — Figure S1. Semaglutide effectively suppresses arrhythmias in murine ventricular cardiomyocytes and multicellular trabeculae. (A) Original registrations of action potential measurements in wildtype murine ventricular cardiomyocytes with an arrhythmia protocol upon vehicle control (V), H2O2, H2O2 + 50 nmol/L semaglutide, and H2O2 + 300 nmol/L semaglutide (top). Percentage of cells showing early afterdepolarizations (EADs, lower left) or delayed afterdepolarizations (DADs, lower right). Expectedly, incidence of EADs and DADs was low in healthy WT cardiomyocytes and required a pathological stimulus (H2O2). Semaglutide effectively lowered the percentage of cells displaying EADs or DADs upon H2O2 and there was no clear difference between 50 or 300 nmol/L semaglutide. Data shown as percentage of cells displaying EADs or DADs. Data tested using Fisher's exact test. (B) Original murine ventricular trabeculae registrations upon baseline (vehicle control), H2O2 + Ca stimulus (to provoke arrhythmias), H2O2 + Ca + 50 nmol/L semaglutide, and H2O2 + Ca + 300 nmol/L semaglutide (left). Mean ± SEM of the arrhythmia score per mouse (right). Expectedly, the arrhythmia score was low at baseline in multicellular murine ventricular preparations and a pathological stimulus (H2O2 + Ca) was required to induce arrhythmogenesis. Semaglutide at 50 or 300 nmol/L effectively reduced the arrhythmia score upon simultaneous H2O2 + Ca exposure. Data shown as mean ± SEM per mouse. Data tested using a mixed‐effects model (overall *p < 0.0001). AP, action potential; DAD(s), delayed afterdepolarizations; EAD(s), early afterdepolarization(s); Sema, semaglutide; V, vehicle control. [file EJHF-27-1315-s001.pdf]
