# Supplementary material for: Cardioprotective effects of semaglutide on isolated human ventricular myocardium
Source: Eur J Heart Fail. 2025 Mar 19;27(7):1315–25. doi: 10.1002/ejhf.3644 (PMC12370581; doi:10.1002/ejhf.3644)
Supplement: Supplementary file 2 — Figure S2. Full statistical reporting for Figure 2E . (A) For better readability of Figure 2E , the full statistical reporting of the multiparameter mixed‐effects model including the Holm‐Sidak post‐test can be found in (B). [file EJHF-27-1315-s003.pdf]

A)

Mixed-effects model

Fixed effects	p-value
Frequency	0.0010
Intervention/Drug	0.0152

Holm-Sidak's multiple comparisons test

	Adjusted P Value
H_2O_2 vs. Vehicle (V)	0.0148
H_2O_2 vs. Sema 50 nM	0.0278
H_2O_2 vs. Sema 100 nM	0.0234
H_2O_2 vs. Sema 300 nM	0.0278

Supplementary figure S2
