# Supplementary material for: Cardioprotective effects of semaglutide on isolated human ventricular myocardium
Source: Eur J Heart Fail. 2025 Mar 19;27(7):1315–25. doi: 10.1002/ejhf.3644 (PMC12370581; doi:10.1002/ejhf.3644)
Supplement: Supplementary file 3 — Supplemental Methods [file EJHF-27-1315-s002.docx]

**Supplemental Methods**

**Cardioprotective effects of semaglutide on isolated human ventricular myocardium**

Thomas Krammer^1^*, Maria J. Baier^1^*, Philipp Hegner^1^*, Tilman Zschiedrich^1^, David Lukas^1^, Matthias Wolf^1^, Christian Le Phu^1^, Vanessa Lutz^1^, Katja Evert^2^, Kostiantyn Kozakov^3^, Jing Li^3^, Andreas Holzamer^3^, Lars S. Maier^1^, Zdenek Provaznik^3^, Donald M. Bers^4^, Stefan Wagner^1#^, Julian Mustroph^1#^

^1^Departments of Internal Medicine II (T.K., M.J.B., P.H., C.LP., D.L., M.W., T.Z., V.L., L.S.M., S.W., J.M.) and ^3^Cardiothoracic Surgery (K.K., J.L., A.H., Z.P.), University Hospital Regensburg, Germany

^2^Institute for Pathology, University of Regensburg, Germany

^4^Department of Pharmacology, University of California at Davis, USA

*T.K., M.J.B, and P.H. contributed equally to this article

^#^S.W. and J.M. contributed equally to this article

**Supplemental methods**

**Isolation of human cardiomyocytes**

Due to the patient collective in our center, all samples were from patients with a Caucasian background (no exclusions due to race or gender). For cardioplegia during transport, LV myocardium (surgery samples and biopsies) was stored in ice-cold Custodiol® solution containing 2 mmol/L butanedione monoxime (BDM). All human and murine cardiomyocyte and tissue experiments used appropriate controls.

Briefly, LV samples were embedded into low-melting-point agarose and cut in 300 µm thick slices in cutting solution containing (in mmol/L): 136 NaCl, 5.4 KCl, 1 MgCl_2_*6 H_2_O, 0.33 NaH_2_PO_4_* 2 H_2_O, 10 glucose, 0.9 CaCl_2_, 30 BDM, 5 HEPES (pH 7.4, 4°C). Myocardial tissue slices were then digested with proteinase bacterial, type XXIV (Sigma, 1 mg/ml) and collagenase, type I (EMD Millipore Corp., 2 mg/ml) in isolation solution containing (in mmol/L): 20 KCl, 10 KH_2_PO_4_, 10 MgCl_2_, 70 glutamic acid, 20 taurine, 10 glucose, 30 BDM, 2 mg/ml BSA (pH 7.3, 37°C). When the tissue was digested and individual myocytes were visible, enzymatic digestion was stopped with stop solution containing (in mmol/L): 20 KCl, 10 KH_2_PO_4_, 10 MgCl_2_, 70 glutamic acid, 20 taurine, 10 glucose, 0.005 CaCl_2_, 10 mg/ml BSA (pH 7.3, 37°C). The digested tissue slices were dissociated with forceps by carefully pulling the fibers apart and calcium concentration was slowly re-adjusted from 5 µmol/L to 1.5 mmol/L (followed by 2.0 mmol/L in the measurement solutions). Cells were plated and settled on laminin-coated chambers for 30 min.

**Isolation of murine cardiomyocytes**

As the murine cardiomyocyte experiments were *in vitro* experiments, no exclusions were performed after inclusion of healthy mice for cell isolation (exclusion criteria for mice relate to animal welfare and were assessed by observation by our animal staff, however, no exclusion criteria were met).

Mice were anaesthetized with isoflurane. After death by cervical dislocation, hearts were quickly excised, mounted on a Langendorff perfusion apparatus and retrogradely perfused with nominally Ca-free solution containing (in mmol/L) NaCl 113, KCl 4.7, KH_2_PO_4_ 0.6, Na_2_HPO_4_x2H_2_O 0.6, MgSO_4_x7H_2_O 1.2, NaHCO_3_ 12, KHCO_3_ 10, HEPES 10, taurine 30, BDM 10, glucose 5.5, phenol-red 0.032 for 4 min at 37°C (pH 7.4). Then, 7.5 mg/mL liberase™ (Roche diagnostics, Mannheim, Germany), trypsin 0.6%, and 0.125 mmol/L CaCl_2_ were added to the perfusion solution. Perfusion was continued for 3-4 min until the heart became flaccid. Ventricular tissue was collected in perfusion buffer supplemented with 5% bovine calf serum, cut into small pieces, and dispersed by repeatedly pipetting until no solid cardiac tissue was left. Ca-reintroduction was performed by stepwise increasing [Ca^2+^] from 0.1 to 1.4 mmol/L (followed by 2.0 mmol/L in the measurement solutions). For measurements, cells were plated and settled (15 min) on laminin-coated superfusion chambers.

**Epifluorescence measurements**

Myocytes were loaded with either the Ca-indicator Fura-2 AM (5 μmol/L, Molecular Probes) or Fluo-4 AM (10 μmol/L, Molecular Probes) for 15 min respectively in darkness. After dye loading with simultaneous exposure to investigational compounds (e.g. semaglutide), preincubation with investigational compounds was continued for a total of 30 min. H_2_O_2_ was added before the start of the measurement protocol. All experiments were performed at 37°C using heated superfusion with Tyrode solution (also containing investigation substances or control). Five minutes of washout and stimulation at 0.5 Hz preceded all experiments. Human myocytes were field-stimulated at 0.5 Hz – 1.0 Hz at steady-state contraction. Murine myocytes were stimulated in a similar manner. Fura-2 fluorescence ratio was calculated by dividing the emission signal obtained by excitation at 340 nm by that obtained at 380 nm, with alternation of excitation at 240 Hz by the IonOptix hyperswitch.

**Confocal measurements**

Cardiomyocytes were loaded with the Ca-indicator Fluo-4 and investigational substances. All experiments were performed at 37°C using heated superfusion with Tyrode solution. Fluorescence measurements for Ca-sparks were performed with a laser scanning confocal microscope (LSM 700 confocal microscope, Zeiss, Germany). Fluo-4 was excited at 488 nm and emitted fluorescence was collected through a 505 nm long-pass emission filter. Fluorescence images were recorded in line-scan mode. Ca-sparks were analyzed and quantified using the ImageJ plugin Sparkmaster (1) with visual confirmation of sparks detected. Ca-spark frequency (CaSpF) was calculated and normalized to scanned myocyte width and scanning interval.

**Patch-clamp experiments**

Patch clamp was performed using ruptured patch (EPC-10 amplifier and Patchmaster software, HEKA) at room temperature. Fast capacitance was compensated in cell-attached configuration, membrane capacitance and series resistance after patch rupture. Access resistance was <7 MΩ. Currents were normalized to membrane capacitance (pA/pF).

Late I_Na_ was recorded as reported previously (2) as the integral of the current between 100 and 500 ms, elicited by square pulses depolarizing the cell to −20 mV (1000 ms duration, 10 pulses) from a holding potential of −120 mV. Pipette solution contained (in mmol/L): 95 CsCl, 40 Cs-glutamate, 10 NaCl, 0.92 MgCl_2_, 5 Mg-ATP, 0.3 Li-GTP, 5 HEPES, 0.03 niflumic acid, 0.02 nifedipine, 0.004 strophanthidin, 1 EGTA, and 0.36 CaCl_2_ (pH 7.2, CsOH). Bath solution contained (in mmol/L) 135 NaCl, 5 tetramethylammonium chloride, 4 CsCl, 2 MgCl_2_, 10 glucose, and 10 HEPES (pH 7.4, CsOH).

Action potentials (APs) and early/delayed afterdepolarizations (EADs/DADs) were recorded as previously described.(3) Intracellular solution consisted of (mmol/L) 120 K-aspartate, 8 KCl, 7 NaCl, 1 MgCl_2_, 10 HEPES, 5 Mg-ATP, 0.3 Li-GTP, 1 EGTA, 0.2 CaCl_2_ (pH 7.2, KOH). Bath solution contained (mmol/L) 140 NaCl, 4 KCl, 1 MgCl_2_, 1 CaCl_2_, 10 glucose, 5 HEPES (pH 7.4, NaOH). APs were continuously elicited by square pulses of 1 nA amplitude and 2-7 ms duration at 1 Hz. EADs and DADs were elicited by stimulation with a train of 30 consecutive current pulses inducing 30 APs at 1 Hz followed by 10 s rest, a second train stimulation of 30 APs at 1 Hz followed by 30 s rest and a final train of 30 APs at 1 Hz. EADs and DADs were analysed during train stimulation and reported as number of cells with EADs/DADs.

**Slice culture and force measurements**

Using the InVitroSys Myo dish tissue cultivation system, 300 µm thick left ventricular myocardial slices were placed between a fixed and a spring wire, allowing the measurement of contraction force over a longer period of time. During cultivation, the slices were continuously stimulated with 30 beats per minute (corresponding to 0.5 Hz) and continuously moved via a rocking system to distribute culture medium homogenously. Contractions were measured at 0, 1, 2, 3, and 4 h timepoints. Baseline was defined as the mean values of contractions at the 0 and 1 h timepoints. Contractions at 2, 3, and 4 h timepoints were normalized to baseline (e.g. figure 3A). Incubation with investigational substances was performed sequentially and run-down (time effects on contractility) excluded by parallel control measurements in separate slices from the same patients. Contractions for figure 3B were vehicle or semaglutide 300 nmol/L, each measured at the 4 h timepoint in separate slices.

**Muscle strip experiments**

Muscle strip experiments were performed as previously reported.(4) Thin papillary muscles from murine right ventricles were isolated in cooled custodiol and 10 mmol/L BDM and connected to a force transducer (World Precision Instruments (WPI), Berlin, Germany) for isometric force recordings and superfused with Krebs–Henseleit solution (mmol/L) 116 NaCl, 5 KCl, 2 NaH_2_PO_4_, 1.2 MgCl_2_, 1.2 Na_2_SO_4_, 20 NaHCO_3_, 10 glucose aerated with 95% O_2_, 5% CO_2_ at a temperature of 37 °C (pH 7.4 with NaOH). Contractions were induced by electrical field stimulation (1 Hz; 5 mA; A385RC, WPI). Ca was increased from initially 0.25 to 2 mmol/L by adding 0.25 mmol/L Ca at 2-minute intervals. After 10 minutes of equilibration, the trabeculae were stretched until reaching the maximum steady-state twitch force to improve comparability. After recording steady state basal contractions, superfusion was switched to solution containing the investigational substances (or control, see above). To stimulate arrhythmias, 100 µM H_2_O_2_ and 3.5 mM Ca were added to the Krebs-Henseleit solution. After an equilibration period of 2.5 minutes, measurements were recorded for 25 minutes. Following this, semaglutide was added, and after another 2.5 minutes of equilibration, a second 25-minute measurement was recorded. Arrhythmias were assessed using the arrhythmia severity score as previously described.(5) For the contractility measurements, the same protocol was followed, but with a physiological Ca concentration of 2 mM. In the vehicle control group, H_2_O_2_ was omitted.

**Western Blot**

Whole-heart homogenates were used. After denaturation for 30 minutes at 37°C in 0.2% β-mercaptoethanol, proteins were separated on 8% sodium dodecyl sulfate polyacrylamide gels, then transferred to a nitrocellulose membrane and incubated with the primary antibody for the GLP-1 receptor (rabbit polyclonal antibody, Thermo Fisher, 26196-1-AP, dilution 1:500) at 4°C overnight. Secondary antibodies were HRP-conjugated donkey anti-rabbit IgG (1:5000, GE Healthcare, NA934) that were incubated for 1 hour at room temperature. For chemiluminescent detection, Immobilon Western Chemiluminescent HRP Substrate (Millipore) was used.

**Supplemental references**

1. Picht E, Zima AV, Blatter LA, Bers DM. SparkMaster: automated calcium spark analysis with ImageJ. *Am J Physiol Cell Physiol*. 2007 Sep;**293**(3):C1073-1081.

2. Neef S, Mann C, Zwenger A, Dybkova N, Maier LS. Reduction of SR Ca(2+) leak and arrhythmogenic cellular correlates by SMP-114, a novel CaMKII inhibitor with oral bioavailability. *Basic Res Cardiol*. 2017 Jul;**112**(4):45.

3. Mustroph J, Wagemann O, Lebek S, Tarnowski D, Ackermann J, Drzymalski M, et al. SR Ca(2+)-leak and disordered excitation-contraction coupling as the basis for arrhythmogenic and negative inotropic effects of acute ethanol exposure. *J Mol Cell Cardiol*. 2018 Mar;**116**:81-90.

4. Sossalla S, Fluschnik N, Schotola H, Ort KR, Neef S, Schulte T, et al. Inhibition of elevated Ca2+/calmodulin-dependent protein kinase II improves contractility in human failing myocardium. *Circ Res*. 2010 Oct 29;**107**(9):1150-1161.

5. Lebek S, Pichler K, Reuthner K, Trum M, Tafelmeier M, Mustroph J, et al. Enhanced CaMKII-Dependent Late I(Na) Induces Atrial Proarrhythmic Activity in Patients With Sleep-Disordered Breathing. *Circ Res*. 2020 Feb 28;**126**(5):603-615.
